# Inappropriate Use of Gastric Acid Suppression Therapy in Hospitalized Patients with *Clostridium difficile*—Associated Diarrhea: A Ten-Year Retrospective Analysis

**DOI:** 10.5402/2012/902320

**Published:** 2012-05-31

**Authors:** Sadat Rashid, Dhyan Rajan, Javed Iqbal, Seth Lipka, Robin Jacob, Valeria Zilberman, Mitanshu Shah, Paul Mustacchia

**Affiliations:** ^1^Department of Gastroenterology, Nassau University Medical Center, East Meadow, NY 11554, USA; ^2^Department of Internal Medicine, Nassau University Medical Center, East Meadow, NY 11554, USA

## Abstract

*Purpose*. The incidence of *Clostridium difficile*-associated diarrhea (CDAD) has steadily increased over the past decade. A multitude of factors for this rise in incidence of CDAD have been postulated, including the increased use of gastric acid suppression therapy (GAST). Despite the presence of practice guidelines for use of GAST, studies have demonstrated widespread inappropriate use of GAST in hospitalized patients. We performed a retrospective analysis of inpatients with CDAD, with special emphasis placed on determining the appropriateness of GAST. *Methods*. A retrospective analysis was conducted at a multidisciplinary teaching hospital on inpatients with CDAD over a 10-year period. We assessed the use of GAST in the cases of CDAD. Data collection focused on the appropriate administration of GAST as defined by standard practice guidelines. *Results*. An inappropriate indication for GAST was not apparent in a majority (69.4%) of patients with CDAD. The inappropriate use of GAST was more prevalent in medical (86.1%) than on surgical services (13.9%) (*P* < 0.001). There were more cases (67.6%) of inappropriate use of GAST in noncritical care than in critical care areas (37.4%) (*P* < 0.001). *Conclusion*. Our study found that an inappropriate use of inpatient GAST in patients with CDAD was nearly 70 percent. Reduction of inappropriate use of GAST may be an additional approach to reduce the risk of CDAD and significantly decrease patient morbidity and healthcare costs.

## 1. Introduction

*Clostridium difficile *(*C. difficile*), a Gram-positive, sporulating, anaerobic bacterium, is the most common cause of nosocomial diarrhea in the United States [1]. The reported cases of *C. difficile-*associated diarrhea (CDAD) have steadily increased over the past decade, with a reported incidence of 0.1–2% in all hospitalized patients [1]. A multitude of risk factors for CDAD have been identified, with the most commonly cited factor being the use of antibiotic therapy [1–3]. Review of literature suggests that the use of gastric acid suppression therapy (GAST) is associated with increasing incidence of CDAD [1–3]. A suggested mechanism for this association is believed to be the increase in gastric pH secondary to GAST, allowing for the survival of the *C. difficile* bacteria and spores [1]. Gastric acid suppression is commonly achieved with the use of proton pump inhibitors (PPIs) and histamine-2 receptor antagonists (H2As). There has been increased use of GAST, especially PPIs in the last decade, correlating with the increased incidence of CDAD [4, 5]. Although an appropriate indication for the use of GAST is often present, inappropriate use of such therapy is frequently observed in an inpatient setting, possibly resulting in an increased incidence of nosocomial CDAD [1, 4, 6].

We performed a retrospective analysis of hospitalized patients with CDAD, with a focus on determining the appropriateness of GAST.

## 2. Methods

A retrospective analysis was conducted at Nassau University Medical Center; a 631-bed multidisciplinary teaching hospital, a part of the North Shore-Long Island Jewish Health System located in East Meadow, New York.

Data was obtained for all inpatients ≥18 years of age from January, 1 2001 to December 31, 2010 who developed CDAD during hospitalization using inpatient medical records after obtaining approval from the hospital's Institutional Review Board (IRB).

Cases were identified using discharge diagnosis of CDAD based on the relevant diagnostic (ICD-9) codes and confirmed by the presence of positive stool *C. difficile* toxins A and B along with associated new onset diarrhea ≥3 days after admission. Patients with prior history of CDAD prior to admission were excluded from the study.

We assessed the use of GAST in the cases of CDAD, as this appears to be an established risk factor for CDAD. GAST was defined as the use of PPIs or H2As after admission to the inpatient service. All patients receiving GAST prior to the first day of admission were excluded from the study. Data collection focused on chart references indicating the reason GAST was administered, via documentation under the “Assessment and Plan” section of the admission progress note. Appropriate uses of GAST for stress ulcer prophylaxis were defined by the American Society of Health-System Pharmacists (AHSP) practice guidelines (Table 1). Appropriate administration of GAST for an established gastrointestinal diagnosis was defined by the Food and Drug Administration's approval guidelines for PPI use (Table 2). If the indication for the use of GAST did not meet the guidelines set by the FDA or AHSP; the use of GAST was deemed inappropriate. Cases of CDAD wherein the indication for GAST was not documented were excluded from the study.

## 3. Statistics

Statistical analysis was performed using the SPSS (version 19). Differences between the patient groups were tested for statistical significance using chi-square analysis. A *P* value of <0.05 was considered statistically significant.

## 4. Results

**Figure 1:**
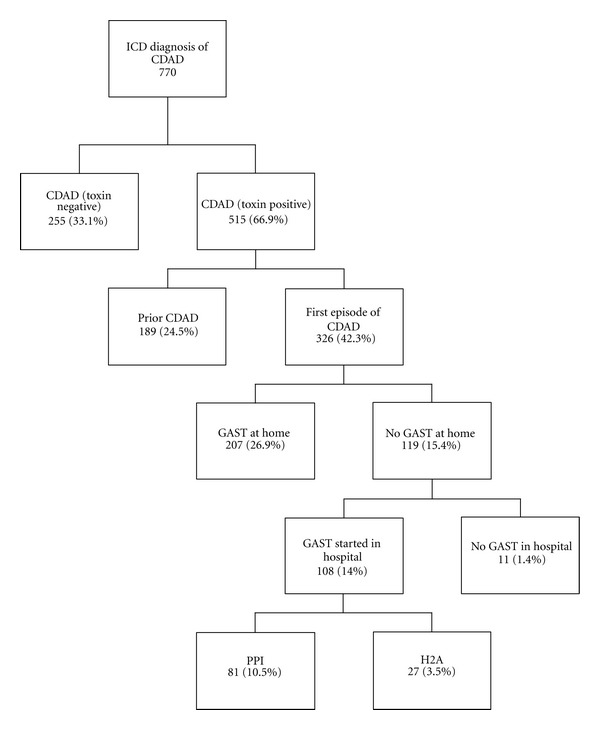
Chart review algorithm for CDAD case selection. ICD: International Classification of Diseases; CDAD: *Clostridium difficile*-associated diarrhea; GAST: gastric acid suppression therapy; PPI: proton pump inhibitor; H2A: histamine-2 receptor antagonist. Note: number in parenthesis indicates percentage of cases.

A total of 770 patients were identified to have a discharge diagnosis of CDAD based on relevant International Classification of Diseases (ICD-9) codes. 515 patients (66.9%) were found to be positive for the *C. difficile *toxin A/B. Of those 515 patients, 326 (63.3%) were noted to have CDAD for the first time. 207 (63.5%) patients that were noted to have CDAD for the first time, were on GAST at home, and of the remaining 119 (36.5%) patients, 108 (90.8%) were started on inpatient GAST and further analyzed in our study (Figure 1).

The mean age of patients with CDAD was 70 years (range 35–97 years). 46 (42.6%) cases were female and 62 (57.4%) were male. The earliest and the latest diagnosis of CDAD after starting inpatient GAST was 4 days and 154 days, respectively; with the average mean duration being 32.4 days (*P* < 0.001). 19.4% of patients who developed CDAD did not receive any antibiotic therapy.

The length of hospital stay after development of CDAD ranged from 4 to 177 days, though this measure did not reach statistical significance, but could have significantly increased the health care costs.

In our study, 81 (75%) patients received inpatient PPI therapy, and the remaining 27 (25%) received H2As as a form of GAST. Of patients receiving PPI therapy, 76.4% received a total daily dose of 40 mg and 23.6% received a daily dose of 80 mg. The dose of H2As used in all of our patients was 40 mg daily. Since our sample size was small, and the majority of patients received a similar dose; a dose correlation with the development of CDAD was beyond the scope of this study.

An appropriate indication for use of inpatient GAST was seen only in 33 (30.6%) cases, while an inappropriate use (Table 3) was seen in 75 (69.4%) cases. The inappropriate use of GAST was more prevalent in medical (86.7%) than surgical (13.3%) services (*P* < 0.001). Inappropriate use of GAST was greater in noncritical care (67.6%) than critical care units (37.4%) (*P* < 0.001).

## 5. Discussion

An association between GAST and CDAD has been proposed in prior studies [1–3, 6, 15–18]. Our study was designed to assess the appropriateness of GAST in patients who developed CDAD.

*Clostridium difficile *infection (CDI) is the most common cause of infectious diarrhea in a nosocomial setting [15]. In United States, approximately 500,000 cases of CDI have been estimated to be present in hospitals and nursing homes [19, 20]. The incidence of CDI in the United States has increased three times over the last decade [20].

A potential mechanism for an association between GAST and CDAD includes an increased pH in the stomach, leading to the growth of pathogenic bacterial flora in the gastrointestinal tract*. C. difficile* spores are acid-resistant but vegetative forms are susceptible to gastric acidity [3, 5, 16, 21]. It has been shown in a hamster model that most of the ingested spores are transformed into the vegetative state within 60 minutes of ingestion, likely in the stomach [21]. It is therefore possible that an increase in gastric pH secondary to GAST may result in germination of sporulated to vegetative forms of *C. difficile *in the stomach, leading to increased colonization with *C. difficile* and subsequent CDAD [21].

The use of GAST for the treatment of gastrointestinal acid-secretory disorders such as gastroesophageal reflux disease (GERD) and peptic ulcer disease (PUD); along with stress ulcer prophylaxis has increased [20]. PPIs have now replaced H2As as the leading GAST due to their more potent acid suppressing properties [20]. Pham et al. found a 140% increase in the use of GAST in patients after admission to the hospital in recent years. The US Pharmaceutical Market Report suggests that over 12.4 billion dollars is spent on the use of PPIs annually [20, 22].

The practice guidelines available for the use of GAST for stress ulcer prophylaxis were published by the American Society of Health-System Pharmacists (Table 1) [7]. The appropriate indications of PPI therapy in the treatment of gastrointestinal acid-secretory disorders have been highlighted by the US Food and Drug Administration (FDA) (Table 2) and are also evident in recent clinical studies [8–14].

Despite the presence of practice guidelines for the use of GAST, multiple studies have demonstrated inappropriate use of GAST in an inpatient setting. Zink et al, and Nardino et al. found an inappropriate indication for GAST prescription in 60% and 72% of all inpatients respectively [23, 24]. The financial impact resulting from the overuse of GAST was illustrated in a study by Heidelbaugh et al. In this study of nearly 1880 inpatients, 22% were found to have an inappropriate indication for GAST, and of those, 54% were discharged home with GAST. The estimated cost of inappropriate GAST from this cohort was nearly $111,000 per year [2].

Our study highlights the fact that the inappropriate use of GAST is common, especially in noncritical care patients, and on medical services.

Although clinicians often view GAST as harmless, its use is not without medical risks and strong financial implications [2, 25–27].

Interventions aimed at improving physician education regarding the appropriate use of GAST may be an additional approach to reduce the risk of CDAD and will significantly decrease healthcare costs.

## Figures and Tables

**Table 1:** ASHP therapeutic guidelines on stress ulcer prophylaxis [7].

Intensive care unit (ICU) patient plus one of the following:
(1) Coagulopathy (i.e., platelet count of <50,000 mm^3^, international normalized ratio (INR) >1.5, or an activated partial thromboplastin time (aPTT) >2 times control)
(2) Mechanical ventilation for >48 hours
(3) History of gastrointestinal ulceration or bleeding within one year of admission
(4) Glasgow coma score of ≤10
(5) Thermal injury to >35% of body surface area
(6) Partial hepatectomy
(7) Multiple trauma (injury severity score of ≥16)
(8) Transplantation perioperatively in the ICU
(9) Spinal cord injury
(10) Hepatic failure
(11) Two or more of the following risk factors: sepsis, ICU stay of greater than one week, occult bleeding lasting at least six days, and high-dose corticosteroids (>250 mg/day of hydrocortisone)

**Table 2:** FDA approved indications for use of proton pump inhibitors [8–14].

(1) Healing of erosive esophagitis
(2) Maintenance of healing of erosive esophagitis
(3) Symptomatic gastroesophageal reflux disease
(4) *Helicobacter pylori* eradication in combination with antibiotics
(5) Short-term treatment of active gastric ulcer
(6) Short-term treatment of active duodenal ulcer
(7) Maintenance of healed duodenal ulcer
(8) Healing of NSAID-Associated gastric ulcer
(9) Risk reduction of NSAID-associated gastric Ulcer
(10) Risk reduction of upper gastrointestinal bleeding in critically Ill patients
(11) Pathological hypersecretory conditions including Zollinger-Ellison syndrome

Abbreviations: FDA, Food and Drug Administration; NSAID, non-steroidal anti-inflammatory drugs.

**Table 3:** Documented inappropriate indications for gastric acid suppression therapy [7–14].

Inappropriate indication	Percent of patients
Indeterminate chest pain	30.6%
Nonspecific abdominal pain	22.6%
Coprescribed with aspirin	14.6%
Coprescribed with low dose steroids	12.0%
Coprescribed with warfarin	10.6%
Coprescribed with antibiotics	5.6%
NPO (nulla per os; nothing by mouth)	2%
Nausea	2%
